# Tooth decay in alcohol and tobacco abusers

**DOI:** 10.4103/0973-029X.80032

**Published:** 2011

**Authors:** Thavarajah Rooban, KM Vidya, Elizabeth Joshua, Anita Rao, Shanthi Ranganathan, Umadevi K Rao, K Ranganathan

**Affiliations:** *Department of Oral and Maxillofacial Pathology, Ragas Dental College and Hospital, Chennai, India*; 1 *TTK Ranganathan Hospital and Research Center, Indra Nagar, Chennai, India*

**Keywords:** Dental caries, India, tobacco, alcohol

## Abstract

**Background:**

Alcohol and tobacco abuse are detrimental to general and oral health. Though the effects of these harmful habits on oral mucosa had been demonstrated, their independent and combined effect on the dental caries experience is unknown and worthy of investigation.

**Materials and Methods:**

We compared 268 alcohol-only abusers with 2426 alcohol and tobacco abusers in chewing and smoking forms to test the hypothesis that various components of their dental caries experience are significantly different due to plausible sociobiological explanations. Clinical examination, Decay, Missing, Filled Teeth (DMFT) Index and Oral Hygiene Index - Simplified were measured in a predetermined format. Descriptive statistics, Chi-square test and one-way ANOVA analysis were done using SPSS Version 16.0.

**Result:**

The mean DMFT were 3.31, 3.24, 4.09, 2.89 for alcohol-only abusers, alcohol and chewing tobacco abusers, smoking tobacco and alcohol abusers, and those who abused tobacco in smoke and smokeless forms respectively. There was no significant difference between the oral hygiene care measures between the study groups. Presence of attrition among chewers and those with extrinsic stains experienced less caries than others.

**Discussion and conclusion:**

The entire study population exhibited a higher incidence of caries experience. Use of tobacco in any form appears to substantially increase the risk for dental caries. Attrition with use of chewing tobacco and presence of extrinsic stains with tobacco use appear to provide a protective effect from caries. The changes in oral micro-flora owing to tobacco use and alcohol may play a critical role in the initiation and progression of dental caries.

## INTRODUCTION

Habitual ‘psychoactive substance (PS) use’ is defined as the repeated use of a PS despite the knowledge of its negative health consequences while ‘PS abuse’ is referred to a pattern of PS use that causes damage to physical or mental health. The common PS use that is of interest to a dentist in India includes alcohol, tobacco and areca nut.[1] It has been reported that the prevalence of dental caries in South India varies with the type of PS use.[1] Dental caries (DC) is a common oral disease that affects any age group and is dependent on a number of factors.

Oral health neglect is a common feature of PS abuse.[2,3] Alcohol has been thought to influence DC via the microbial oxidation of ethanol in saliva in alcohol abusers resulting in the formation of acetaldehyde that inhibits the cariogenic oral flora. Alcohol enhances fluoride release from certain restorative materials.[4] Nicotine, a major constituent of tobacco, is known to limit the proliferation of *Streptococcus viridians*.[5] On the contrary, sugar-laced chewing tobacco extracts have been shown by *in vitro* evidence for stimulated growth of *S. mutans* and *S. sanguis*.[6]

It is considered that frequent chewing of areca nut confers a protection against DC. Areca nut by itself lacks ingredients that have cariostatic properties. The extrinsic stain formed by the chronic habit acts as a laminate preventing adherence and colonization of the cariogenic microbes. The gritty consistency of the areca nut mediates a mechanical cleansing activity eliminating the food debris.[7] Repeated chewing stimulus results in an increased salivary flow rate that also aids in the removal of organisms and food debris. The tannins in this bolus have antimicrobial properties. Attrition in chewers makes the teeth surface smooth and reduces the risk of pit and fissure caries. The sclerosis of dentin by repeated masticatory trauma renders the dentin resistant to the microbial invasion.[7] The addition of lime alters the pH of the oral cavity making it unsuitable for the cariogenic organisms to survive.[8] Moreover, the salivary flow rate and pH have been shown to vary with the type of areca nut and tobacco chewed.[9]

The understanding of the influence of PS on DC will help to limit the overall oral disease burden as well have a huge impact on the socioeconomic component of the dental disease burden in this vulnerable population. In India, the most common PSs abused are alcohol and tobacco.[1–3] Given the large percentage of Indian population abusing PSs, It is still unclear how different PS use influences the overall DC experience in the Indian population. Hence this study was undertaken with the objective to evaluate the effect of different PS use in different combination, for understanding the association between PS uses and different components of the dental caries experience. We hypothesize that DC is influenced by the type of PS use.

## MATERIALS AND METHODS

A retrospective study of consecutive first-visit persons who attended the dental clinical care facilities over a period of seven years (June 2002 to May 2009) at TTK Hospital, Chennai, India formed the study group. It serves the local district population and also people from the adjoining districts and states including Karnataka, Andhra Pradesh and Kerala, and its valuable services are recognized by several forums including the United Nations Office of Drug and Crime, Regional Office of South Asia by deeming it as a training institute for Non-Governmental Oragnaizationin the prevention and treatment of PS use. Ragas Dental College and Hospital, Chennai caters to the oral hygiene and dental treatment needs of the patients enrolled at TTK Hospital.

Trained physicians and dental surgeons calibrated and examined the patients. Their clinical findings were recorded in a predetermined format, which included detailed recording of the patients’ habits (alcohol and tobacco (with/without areca nut)) as per earlier published protocols and clinical observations including Oral Hygiene Index-Simplified (OHI-S) and Dental Caries, Missing, Filled Tooth (DMFT) index.[1] Tobacco use was measured as pack (ten’s) years. Smoking tobacco pack years were calculated as published in the literature and smokeless tobacco (2 gm per pack) used per year as pack years.

Only dentate subjects were enrolled for the study. Based on their PS habits, the study group was broadly divided into four groups without any overlap. They were alcohol-only abusers (A), alcohol and smoking tobacco abusers (AS), alcohol and chewing tobacco abusers (AC) and smoking, chewing tobacco with alcohol abusers (ASC). Presence of attrition and extrinsic stain (< two-thirds of any surface in any teeth) were noted. Occasional tobacco users were excluded. For the present study, tobacco use was considered as abuse when the subject used any form and quantity of tobacco continuously for three months. Alcohol abuse was considered as per standard definitions.[1]

Data were entered and analyzed using the Statistical Package for Social Services, Version 16.0 (SPSS Inc., IL, USA). Descriptive statistics were presented for all variables. Pearson’s Chisquare test was performed to determine the significance of associations between demographic characters and habits. Odds ratios (ORs) and 95% confidence intervals (CIs) were calculated to find the association between various habits with DMFT and DC. One-way ANOVA was employed to find the difference in the mean of DC experience among the groups. *P* value < 0.05 was considered to be statistically significant.

## RESULTS

There were 2694 patients considered for the study. The demographic details of the study groups are detailed in Table 1. There were 2689 males (99.81%) with a mean age of 38.49±8.27 years (18 to 70 years) with the majority of them belonging to the 36 to 40 years age group (24.1%). The mean age and age group across study groups were statistically significant. The majority of the study population were married and education differed significantly across study groups (*P* =0.018). The duration of tobacco habit was as follows: smoking tobacco use ranged from three months to 41 years with a mean of 13.5±8.12 years, chewing tobacco (in processed forms), six months to 40 years with a mean of 7.48±5.6 years while raw tobacco use was for a period of 13.3±8.95 years. The mean pack years for alcohol abusers chewing tobacco was 265.54, for smoking alcohol abusers was 588.81 and for those alcohol abusers who smoked and chewed tobacco was 575.08.

**Table 1:** Demographic characteristics of the study population (*n* = 2694)

	Alcohol (*n =*268) (%)	Alcohol + Chewing (*n*=691)*n*(%)	Alcohol + Smoking (*n* = 1056)*n*(%)	Alcohol + Chewing + Smoking (*n* = 679)*n*(%)	*P*value
Gender					0.613
Males	268 (100)	689 (99.7)	1055 (99.9)	677 (99.7)	
Females		2 (0.3)	1 (0.1)	2 (0.3)	
Mean age (in years)	42.8±8.74	37.28±7.16	40.51±8.3	34.88±7.33	0.000**
Age group					0.000**
Below 20	0	2 (0.29)	1 (.09)	3 (.44)	
21-25	4 (1.49)	18 (2.6)	21 (1.99)	48 (7.07)	
26-30	20 (7.46)	103 (14.91)	98 (9.28)	154 (22.68)	
31-35	32 (11.94)	172 (24.89)	196 (18.56)	179 (26.36)	
36-40	57 (21.27)	194 (28.08)	239 (22.63)	160 (23.56)	
41-45	58 (21.64)	107 (15.48)	211 (19.98)	77 (11.34)	
46-50	42 (15.67)	66 (9.55)	151 (14.3)	38 (5.6)	
51-55	32 (11.94)	22 (3.18)	93 (8.81)	11 (1.62)	
55-60	18 (6.72)	5 (.72)	41 (3.88)	9 (1.33)	
above 61	5 (1.87)	2 (0.29)	5 (0.47)	0	
Marital status					0.000**
Married	252 (94.4)	605 (87.6)	952 (90.2)	530 (78.3)	
Unmarried	15 (5.6)	82 (11.9)	98 (9.3)	145 (21.4)	
Separated	0	4 ((0.6)	5 (0.5)	2 (0.3)	
Religion					0.067
Hindu	248 (92.5)	646 (93.5)	940 (89.2)	607 (89.4)	
Christian	13 (4.9)	24 (3.5)	79 (7.5)	47 (6.9)	
Muslim	7 (2.6)	20 (2.9)	34 (3.2)	25 (3.7)	
Sikh	0	1 (0.1)	1 (0.1)	0	
Education					0.018*
No education	8 (3)	33 (4.8)	42 (4)	28 (4.1)	
Primary school	47 (17.5)	105 (15.2)	127 (12)	109 (16.1)	
Secondary school	99 (36.9)	307 (44.4)	431 (40.8)	259 (38.1)	
College	114 (42.5)	246 (35.6)	456 (43.2)	283 (41.7)	
Alcohol units	100.41±53.54	103.73±57.31	108.31±62.83	115.41±69.19	0.001**
Duration of alcohol use	11.79±8.46	10.01±6.62	12.20±8.1	9.93±6.34	0.000**
At least 1 caries	152 (56.7)	428 (61.9)	620 (58.7)	378 (55.7)	0.113
At least 1 missing	106 (39.6)	236 (34.2)	511 (48.4)	222 (32.7)	0.000**
At least 1 filling	20 (7.5)	46 (6.7)	88 (8.3)	44 (6.5)	0.432
Mean remaining teeth	26.56±3.08	27.06±1.99	26.2±3.24	27.16±1.65	0.000**
Mean dental caries	1.72±2.22	2.18±2.56	2.06±2.62	1.91±2.65	0.049*
Mean missing	1.44±3.08	0.94±1.99	1.8±3.24	0.84±1.66	0.000**
Mean filling	0.15±0.63	0.13±0.57	0.24±1.16	0.14±0.67	0.03*
Mean DMFT	3.31±3.79	3.24±3.44	4.09±4.45	2.89±3.42	0.000**
Mean OHI	1.77±0.99	1.87±0.97	1.84±0.96	1.93±0.95	0.075

* *P*<0.05 - Statistically significant

** *P*≤.0.001 - High statistical significance

Across study groups, prevalence of at least one DC was not statistically significant (*P*=0.113), while at least one missing tooth (*P*=0.000) was significant. The mean difference in DMFT, OHI-S, DC, missing and filled teeth across study groups was significant. Mean years of alcohol abuse and units of alcohol consumed per week were significantly different across the study groups (*P* =0.001 and 0.000 respectively).

**Figure 1:**
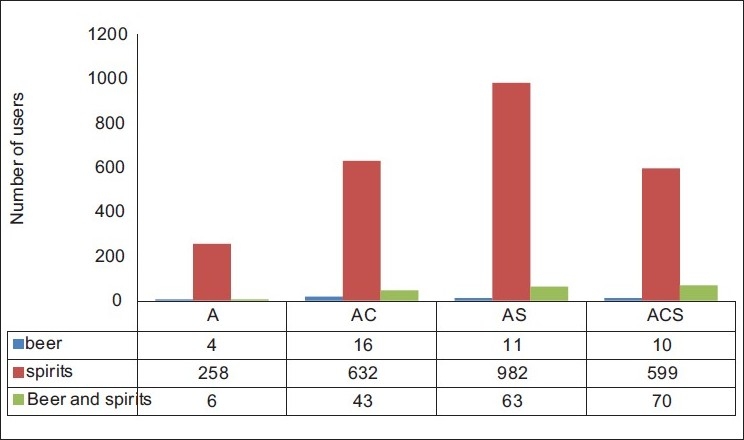
Type of alcohol user in the study population

Figure 1 depicts the type of alcohol used by the study group. Table 2 depicts the oral hygiene measures adopted by the study population and was not significantly different across groups. Outcome of oral hygiene measures was measured as caries experience. Material and methods used for oral hygiene were significantly associated with OHI-S and DMFT as well as missing teeth [Table 3].

**Table 2:** Oral hygiene measures in the study population (*n* = 2694)

	Alcohol (*n* = 268)*n*(%)	Alcohol + Chewing (*n* =691)*n*(%)	Alcohol + Smoking (*n* = 1056)*n*(%)	Alcohol + Chewing + Smoking (*n* = 679)*n*(%)	*P* value
Cleaning material					0.329
Toothpaste	254 (94.78)	666 (96.38)	1023 (96.88)	660 (97.2)	
Toothpowder	10 (3.73)	20 (2.89)	21 (1.99)	16 (2.36)	
Others	4 (1.49)	5 (0.72)	12 (1.14)	3 (0.44)	
Frequency					0.094
Once	226 (84.3)	600 (86.8)	887 (84)	597 (87.9)	
More than once	42 (15.7)	91 913.2)	169 (16)	82 (12.1)	
Cleaning method					0.149
Toothbrush	259 (97)	682 (98.8)	1024 (97.7)	665 (98.1)	
Fingers	7 (2.6)	7 (1)	24 (2.3)	13 (1.9)	
Others	1 (0.4)	1 (.1)	0	0	

**Table 3:** Oral hygiene measures compared with outcome variables for ever dental caries experience

		DMFT	OHI-S	Dental caries	Missing teeth	Filled teeth
Material used for oral hygiene measures						
A	Toothpaste	3.30±3.78	1.76±0.98	1.72±2.17	1.43±3.12	0.16±0.65
	Toothpowder	2.60±3.57	2.19±1.27	1.10±1.91	1.40±2.50	0.10±0.32
	Others	5.25±4.99	1.50±0.58	3.25±5.25	2.00±1.83	0
AC	Toothpaste	3.24±3.45	1.84±0.95	2.19±2.56	0.93±1.99	0.12±0.58
	Toothpowder	2.90±3.11	2.46±1.31	1.70±2.52	1.00±1.56	0.20±0.52
	Others	5.20±3.56	2.56±1.18	3.20±3.11	2.00±3.94	0
AS	Toothpaste	4.00±4.36	1.83±0.96	2.02±2.58	1.74±3.16	0.24±1.18
	Toothpowder	7.86±6.65	2.14±1.16	3.52±3.97	4.33±4.86	0
	Others	5.33±5.19	1.88±0.83	2.58±2.57	2.75±4.67	0
ACS	Toothpaste	2.89±3.43	1.91±0.94	1.90±2.65	0.85±1.67	0.14±0.68
	Toothpowder	2.50±3.16	2.74±1.22	1.88±2.28	0.63±1.36	0
	Others	4.67±3.06	2.87±1.37	4.33±3.51	0.33±0.58	0
	*p*value	0.021*	0.000**	0.117	0.02*	0.388
Frequency of oral hygiene measures						
A	Once	3.38±3.84	1.72±0.93	1.8±2.23	1.45±3.17	0.14±0.6
	more than once	2.91±3.48	2.06±1.24	1.29±2.16	1.38±2.58	0.24±0.79
AC	Once	3.39±3.45	1.86±0.97	2.29±2.58	0.98±2.06	0.12±0.51
	more than once	2.26±3.2	1.89±0.98	1.44±2.32	0.67±1.45	0.15±0.89
AS	Once	3.96±4.22	1.86±0.96	2.00±2.54	1.74±3.07	0.22±1.09
	more than once	4.81±5.45	1.70±0.97	2.35±3.02	2.11±4	0.35±1.46
ACS	Once	2.88±3.41	1.93±0.92	1.90±2.63	0.83±1.66	0.15±0.71
	more than once	2.92±3.53	2.00±1.21	1.98±2.75	0.89±1.62	0.05±0.22
	*p* value	0.597	0.716	0.508	0.309	0.211
Method of oral hygiene measures						
A	Brush	3.22±3.72	1.76±0.98	1.70±2.17	1.37±3.05	0.15±0.64
	Finger	6.71±5.09	2.17±1.37	2.71±3.82	3.71±3.59	0.29±0.49
	Neem stick	4	2	0	4	0
AC	Brush	3.23±3.44	1.86±0.96	2.18±2.56	0.93±1.98	0.13±0.58
	Finger	3.14±2.73	2.00±1.24	2.43±2.64	0.71±1.11	0
	Neem stick	5	3.10	5	0	0
AS	Brush	4.04±4.41	1.83±0.95	2.04±2.61	1.75±3.18	0.24±1.18
	Finger	6.83±5.4	2.27±1.13	2.79±2.86	4.00±4.95	0.04±0.2
ACS	Brush	2.88±3.43	1.92±0.95	1.90±2.65	0.84±1.66	0.14±0.68
	Finger	3.31±3.35	2.35±1.01	2.31±2.84	1.00±1.47	0
	*P* value	0.002*	0.011*	0.251	0.000^**^	0.606

A - alcohol, C - chewing, S - smoking

* *P<05, significant

** *P* = 0.000 – high significance

Bivariate logistic regression between DMFT greater than or equal to 1 and 0, revealed that marital status (OR 1.217, 95%CI- 0.95 – 1.560, *P* = 0.122), religion (OR 1.03; 95%CI 0.84 – 1.25, *P* = 0.797), education (OR 1.03; 95%CI – 0.93 – 1.15; *P* = 0.54) were not significant while age group (OR 1.07; 95%CI – 1.01 – 1.13; *P* = 0.02) and occupation (OR 1.04; 95%CI 1.008 – 1.066; *P* = 0.011) were significant. On further analysis of age group and occupation, none of the individual subgroups had ORs that were statistically significant.

Similarly, for DC greater than or equal to 1 and 0, bivariate logistic regression revealed that marital status (OR 1.032, 95%CI- 0.827 – 1.289, *P* = 0.778), occupation groups (OR 1.023; 95%CI 0.998 – 1.049; *P* = 0.075), religion (OR 1.039; 95%CI 0.865 – 1.247, *P* = 0.686), education (OR 1.012; 95%CI – 0.919 – 1.114; *P* = 0.81) were insignificant while age group (OR 0.924; 95%CI – 0.879 – 0.971; *P* = 0.002) was significant. On further analysis of age group, none of the individual subgroups had statistically significant OR.

**Figure 2:**
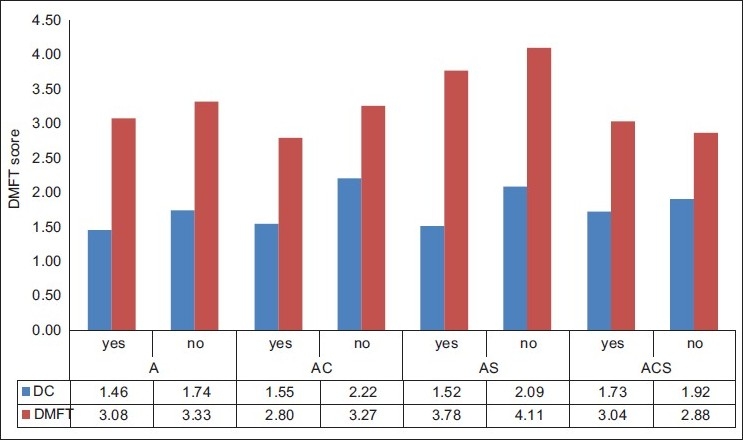
Graph showing mean DMFT and dental caries in study group with attrition and without attrition

There was a statistical significance between the prevalence of DMFT, missing teeth and filled teeth between chewers and non-chewers while filled teeth was only significant between those with and without pouching habit [Figure 2]. There was a statistically significant difference in the caries experience between those with and without attrition with a *P* value of 0.021 [Table 4]. The mean DC in patients having attrition was 1.56 while for patients with no attrition it was 2.05.

**Table 4:** Chewing and pouching and the study population

		DMFT	Dental Caries	Missing	Filled
Chewing					
Yes	AC	3.28±3.61	2.19±2.64	0.95±2.15	0.14±0.63
	ACS	2.81±3.43	1.87±2.66	0.83±1.67	0.11±0.52
No	A	3.32±3.79	1.72±2.22	1.44±3.08	0.15±0.63
	AC	3.15±2.99	2.15±2.37	0.9±1.56	0.1±0.41
	AS	4.09±4.45	2.06±2.62	1.80±3.24	0.24±1.16
	ACS	3.29±3.37	2.10±2.57	0.89±1.57	0.3±1.17
	*P* value	0.000**	0.98	0.000**	0.01*
Pouching					
Yes	AC	3.14±2.97	2.16±2.37	0.87±1.54	0.10±0.42
	ACS	3.22±3.29	2.12±2.54	0.83±1.51	0.27±1.11
No	A	3.31±3.78	1.72±2.22	1.44±3.08	0.15±0.63
	AC	3.29±3.62	2.19±2.64	0.96±2.16	0.13±0.63
	AS	4.09±4.45	2.06±2.62	1.80±3.24	0.24±1.16
	ACS	2.82±3.45	1.87±2.67	0.84±1.68	0.11±0.53
	*P* value	0.107	0.348	0.001**	0.779
Attrition					
Yes	A	3.08±3.06	1.46±1.91	1.42±1.77	0.21±0.72
	AC	2.8±3.18	1.55±2.17	1.05±1.6	0.20±0.56
	AS	3.78±4.5	1.52±2.37	2.22±3.25	0.05±0.28
	ACS	3.04±2.85	1.73±2.66	1.23±1.99	0.08±0.39
No	A	3.33±3.85	1.74±2.25	1.44±3.18	0.15±0.62
	AC	3.27±3.45	2.22±2.58	0.93±2.01	0.12±0.57
	AS	4.11±4.45	2.09±2.64	1.77±3.24	0.25±1.19
	ACS	2.88±3.44	1.92±2.65	0.82±1.64	0.14±0.68
	*P* value	0.522	0.021**	0.113	0.39
Extrinsic stain					
Yes	A	3.27±3.15	2±2.43	1.12±2.04	0.15±0.64
	AC	3.07±2.98	2.12±2.34	0.83±1.64	0.13±0.52
	AS	3.76±3.7	1.92±2.37	1.64±2.59	0.2±0.76
	ACS	3.02±3.12	1.89±2.34	0.97±1.85	0.16±0.73
No	A	3.36±4.55	1.32±1.82	1.88±4.1	0.16±0.63
	AC	3.5±4.03	2.27±2.87	1.11±2.43	0.12±0.65
	AS	4.46±5.12	2.21±2.87	1.97±3.82	0.28±1.48
	ACS	2.74±3.74	1.93±2.97	0.69±1.39	0.12±0.59
	*P* value	0.019**	0.359	0.021**	0.393

A - alcohol, C - chewing, S - smoking

* statistically significant

** highly significant

Table 5 depicts the results of one-way ANOVA for DMFT, DC, missing and filled teeth. The means of filled and dental caries-affected teeth were significantly different across the group. Pack years did not influence the DMFT scores.

**Table 5:** One-way ANOVA of mean dental caries experience in study group with confidence interval and significance

		N	Mean	Std. Deviation	95% Confidence interval for mean		*P* value
					Lower bound	Upper bound	
DMFT	A	268	3.306	3.78491	2.8508	3.7612	0.000**
	AC	691	3.2417	3.43693	2.985	3.4984	
	AS	1056	4.0938	4.45043	3.825	4.3625	
	ACS	679	2.8881	3.42072	2.6303	3.1458	
Dental caries	A	268	1.7164	2.22216	1.4492	1.9837	0.049*
	AC	691	2.1809	2.56238	1.9895	2.3723	
	AS	1056	2.0578	2.62425	1.8993	2.2162	
	ACS	679	1.9102	2.64729	1.7107	2.1096	
Missing teeth	A	268	1.4366	3.07821	1.0664	1.8068	0.000**
	AC	691	0.9363	1.99172	0.7876	1.0851	
	AS	1056	1.8002	3.24027	1.6045	1.9958	
	ACS	679	0.8395	1.6552	0.7147	0.9642	
Filled teeth	A	268	0.153	0.63224	0.0769	0.229	0.030*
	AC	691	0.1245	0.57395	0.0816	0.1673	
	AS	1056	0.2367	1.15968	0.1667	0.3068	
	ACS	679	0.1384	0.6707	0.0879	0.189	

A - alcohol, C - chewing, S - smoking

* statistically significant

** highly significant

## DISCUSSION

Dental caries is a multi-factorial, microbial, universal disease affecting all geographic regions, races, both the sexes and all age groups. The prevalence of DC is generally estimated at the ages of 5, 12, 15, 35–44 and 65–74 years for global monitoring of trends and international comparisons. Prevalence of DC in India in these age groups is 56.72, 47.39, 49.59, 42.24 and 70.65 respectively. DMFT in the same ages are 2.1, 1.6, 1.37, 1.39 and not recorded for the 65-74 years.[10]

Reports of DC among PS users from various parts of the globe have been documented. In a survey of hospitalized alcoholic patients in Wyoming, USA, alcohol abusers had a three times higher permanent tooth loss than the USA’s national average for corresponding ages.[11] A smaller group of alcoholics in Maryland, USA also had a higher number of missing teeth.[12] In a case-control study of 85 volunteer Finnish alcoholics, there were significantly fewer teeth and more remaining teeth with DC.[13] Use of tobacco and or areca nut[7,8] in various forms and its interaction is known to cause abnormality in salivary pH, flow rate[8] as well as the oral micro-flora[5,6] thereby influencing the initiation and progression of DC. Mean age, DC, DC point prevalence and DMFT in the entire study population was 38.49 years, 2.02±2.6, 58.6% and 3.49±3.93 respectively. These values are comparatively higher than the Indian national average of DC - 42.24% and DMFT of 1.39. This indicates that PS use has a larger role to play in poor oral health. This has been in accordance with previous reports such as those of Dasanayake *et al*., from London.[4] In our earlier reports from this part of India, PS use has been documented to have DC experience varying with various type of PS. However, the DC experience has not been studied in detail in those reports.[1–3]

In the present study, there was a significant statistical difference when the mean remaining teeth, DC, filled teeth and DMFT were compared across the various types of PS abuse [Table 1]. This indicates that the type of PS abused would probably influence the DC experience and oral hygiene status. About 95% of all subjects in each study group used toothpaste, more than 80% of them brushed once a day and more than 97% used a toothbrush to maintain oral hygiene. The oral hygiene measures were not significantly different between the study groups. On the contrary, the type of PS abuse differed with respect to current DMFT status. The brushing material (toothpaste/toothpowder/others) used and mode of oral hygiene care (toothbrush/fingers/others) had a significant difference in terms of current DMFT and OHI-S scores. This finding also explains that the type of PS would probably be a major factor in determining the DC, DMFT as well as OHI-S. As Table 2 indicates, the method of oral hygiene care used by the subjects in the present study, did not significantly differ among study groups indicating that the PS abused is an important factor that differed in the study population. Though tobacco abuse was prevalent for longer periods among the study groups, as indicated by the higher mean duration, it was not contributory.

Tobacco usage in any form immediately increases salivary flow, but the effect of long-term use is poorly understood. The pH of saliva tends to rise during smoking tobacco, which in the long term reduces marginally. There are reports of increasing concentration of thiocynate in saliva, probably from the smoked form of tobacco.[14] Lower cystatin activities have been reported in tobacco smokers. Cystatins are believed to contribute to balanced oral health by inhibiting certain proteolytic enzymes.[15] There have been contradictory reports of DC in tobacco smokers. A few studies show a higher incidence of DC in smokers[16] while some show decreased activity of *Streptococci* and other oral commensals[5] and other studies failed to show any differences.[15] Our study is in concurrence with previous findings of increased incidence of DC among smokers.[4,17]

Offenbacher and Weathers[18] reported on the dental effects of smokeless tobacco use among school-aged males from Georgia. In their study, DMFT scores for smokeless tobacco users with gingivitis were higher than for those who did not use smokeless tobacco and did not have gingivitis. From their findings they concluded that the presence of gingivitis was an indicator of oral hygiene and that poor oral hygiene was a cofactor with smokeless tobacco use in the development of dental caries.[18] However, the smokeless tobacco in Western countries[19] and several areca nut preparations in India[17] contained varying amount of sugars which could be responsible for root caries rather than coronal caries as well as an increased amount of gingival recession in smokeless tobacco users.[19] In the present study, the increased incidence of DC in the groups that used tobacco, chewing (2.18), smoking (2.06) or both (1.91) in addition to alcohol as compared to the alcohol-only usage group (1.72), experienced higher DC. This finding supports the fact that tobacco in any form increases the risk of DC.

As indicated in Tables 1, 5, the higher incidence of missing teeth due to DC, particularly in alcoholic smokers is another indicator of the synergistic effect of tobacco use and poor oral hygiene that has been reported earlier.[17] Analysis of chewing and pouching habits [Table 4] confirm the fact that smokeless tobacco with/without areca nut when chewed causes less DC than when pouched. These findings were in agreement with the reports of Moller *et al*.[7] Similarly, in those cases who had attrition, prevalence of DC was lower. This could be due to the fact that attrition could lower the grooves and pits, which probably play a major role in the initiation of DC.[7]

As observed in Table 4, DMFT between those with significant extrinsic stain and without it were not significantly different while the incidence of DC classified on the presence and absence of attrition had a statistically significant difference. These findings reiterate the fact that chewing forms could cause attrition, and DC in such situations are less. Moreover, extrinsic stains could act as a protective laminated covering and aid in prevention of DC.[7,8] In the present study, the difference between the incidence of DMFT score and missing teeth was significantly higher in subjects with > two-thirds of surface with extrinsic stains than with others [Table 4].

The interaction of oral flora with PS abuse has not been reported in the literature to the best of our knowledge. However, a smaller sample size has been used to report the changes in oral microflora with PS use, especially use of chewing tobacco.[20] It has been showed that use of chewing tobacco decreased the colony-forming units’ count of *Lactobacillus, Prevotella* and *Porphyromonas* species and increased *Fusobacterium* species.[20] In our study, the mean dental caries experience among the types of PS abuse, significantly different, in terms of caries experience, could have probably been due to the postulated decrease in the normal oral microbial flora as a result of PS use.

Several limitations of the study design have to be considered when interpreting the findings from this present study. Data on tobacco use are based on the survey participants’ self-reported information. This carries an inherent potential for bias. However, several such cross-sectional surveys of tobacco use by adults, have shown that such studies have relatively low rates of misreporting.[21] The data used in this study were cross-sectional in nature. Therefore, establishing the temporal sequence of exposure and DC— that is, use of chewing tobacco preceded DC development is practically impossible. Non-use of radiographic diagnostic aids would have understated the actual incidence of DC.

## CONCLUSION

The present study, to the best of our knowledge, is the first study to document and compare the till date experience of dental caries and compare it across various commonly abused PSs, viz., alcohol, chewing tobacco and smoking tobacco forms. Poorer OHI observed among PS users indicates the physical neglect of oral hygiene measures and warrants a detailed exploration of the phenomenon. The higher prevalence of dental caries indicates the fact that dentists should be a part of the team that treats the PS abuse and this would help the patients to greatly improve their quality of life after successful cessation of PS abuse.
